# Multistate outbreak of *Salmonella* Poona infections associated with imported cucumbers, 2015–2016

**DOI:** 10.1017/S0950268819001596

**Published:** 2019-09-12

**Authors:** M. Laughlin, L. Bottichio, J. Weiss, J. Higa, E. McDonald, R. Sowadsky, D. Fejes, A. Saupe, G. Provo, S. Seelman, J. Concepción-Acevedo, L. Gieraltowski, The Outbreak Investigation Team

**Affiliations:** 1 Division of Foodborne, Waterborne, and Environmental Diseases, Centers for Disease Control and Prevention, Atlanta, GA, USA; 2 Arizona Department of Health Services, Phoenix, AZ, USA; 3 Maricopa County Department of Public Health, Phoenix, AZ, USA; 4 California Department of Public Health, Los Angeles, Richmond and Sacramento, CA, USA; 5 County of San Diego Health and Human Services Agency, San Diego, CA, USA; 6 County of San Diego Department of Environmental Health, San Diego, CA, USA; 7 Nevada Division of Public and Behavioral Health, Carson City, NV, USA; 8 Utah Department of Health, Salt Lake City, UT, USA; 9 Montana Department of Public Health and Human Services, Helena, MT, USA; 10 Minnesota Department of Health, St. Paul, MN, USA; 11 Minnesota Department of Agriculture, St. Paul, MN, USA; 12 Alaska Division of Public Health, Juneau, AK, USA; 13 United States Navy, San Diego, CA, USA; 14 Center for Food Safety and Applied Nutrition, Food and Drug Administration, College Park, MD, USA

**Keywords:** Enteric bacteria, Food-borne infections, Food safety, Salmonellosis

## Abstract

We investigated a large multistate outbreak that occurred in the United States in 2015–2016. Epidemiologic, laboratory, and traceback studies were conducted to determine the source of the infections. We identified 907 case-patients from 40 states with illness onset dates ranging from July 3, 2015 to March 2, 2016. Sixty-three percent of case-patients reported consuming cucumbers in the week before illness onset. Ten illness sub-clusters linked to events or purchase locations were identified. All sub-clusters investigated received cucumbers from a single distributor which were sourced from a single grower in Mexico. Seventy-five cucumber samples were collected, 19 of which yielded the outbreak strain. Whole genome sequencing performed on 154 clinical isolates and 19 cucumber samples indicated that the sequenced isolates were closely related genetically to one another. This was the largest US foodborne disease outbreak in the last ten years and the third largest in the past 20 years. This was at least the fifth multistate outbreak caused by contaminated cucumbers since 2010. The outbreak is noteworthy because a recall was issued only 17 days after the outbreak was identified, which allowed for the removal of the contaminated cucumbers still available in commerce, unlike previous cucumber associated outbreaks. The rapid identification and response of multiple public health agencies resulted in preventing this from becoming an even larger outbreak.

## Introduction

*Salmonella* is a leading bacterial cause of gastroenteritis in the United States and continues to be a significant public health problem [1], causing an estimated one million illnesses and 400 deaths annually [2]. Multistate foodborne outbreaks have become more frequent, due in part to increases in national and international food distribution routes, which can contribute to the spread of contaminated food products [3].

*Salmonella enterica* serovar Poona is a relatively rare serotype and is responsible for one percent of human *Salmonella* illnesses reported in the United States, according to the national molecular-subtyping network for foodborne disease surveillance (PulseNet) [4]. Past outbreaks of *Salmonella* Poona infections have been associated with fresh cantaloupe and pet turtles [4,5]. Historical data from PulseNet indicate that approximately five reported illnesses per month are expected from *Salmonella* Poona pulsed-field gel electrophoresis (PFGE) pattern JL6X01.0018 (the pattern that was eventually determined to be the primary outbreak strain) in the summer months in the United States. This expected number drops to one reported illness per month during the winter months.

On August 18, 2015, a cluster of 18 *Salmonella* Poona isolates from 13 states was detected by PulseNet; these isolates were indistinguishable by PFGE analysis. In this report, we describe the outbreak investigation, identification of the outbreak vehicle and implementation of control measures.

## Methods

### Outbreak identification and case definition

Cases were identified using PulseNet. Local and state public health laboratories received *Salmonella* isolates from clinical illnesses for PFGE subtyping with standardised methods [6]. These public health laboratories submitted PFGE patterns to PulseNet. The initial case definition was a laboratory-confirmed infection with *Salmonella* Poona with PFGE *Xba*I pattern JL6X01.0018 with illness onset on or after July 3, 2015. During August and September 2015, PulseNet detected four additional PFGE clusters of *Salmonella* Poona that appeared to be related based on the PFGE patterns, cucumber consumption reported by case-patients and distribution and timing of the infections. Based on these similarities, the five PFGE patterns were combined, and the revised case definition became: a laboratory-confirmed *Salmonella* Poona infection with illness onset date from July 3, 2015 to March 2, 2016, with any of the five outbreak strains (JL6X01.0018, JL6X01.0129, JL6X01.0375, JL6X01.0778 or JL6X01.0784).

### Hypothesis generation

Case-patients were interviewed with a standard questionnaire, collecting exposure information for more than 300 possible food, water and environmental exposures in the week before illness onset. Focused questionnaires were deployed once hypotheses about the outbreak source were identified. The focused questionnaire collected detailed product information for a limited number of leading hypotheses such as brand, purchase location and purchase date. Food exposures reported by case-patients were compared to the 2006–2007 FoodNet Population Survey, a survey of healthy people, to determine if case-patients consumed any foods more often than expected, compared to the general population [7]. Sub-clusters of case-patients were also identified. An illness sub-cluster is defined as two or more people not living in the same household who reported eating at the same restaurant location, attending a common event or shopping at the same location of a grocery store in the week before becoming ill.

### Traceback investigations

Local and state health and agriculture departments and the United States Food and Drug Administration (FDA) conducted traceback investigations of the likely food vehicle, collected food samples and inspected identified facilities for potential sources of contamination. Local, state, Centers for Disease Control and Prevention (CDC) and FDA officials selected a subset of illness sub-clusters and one fatal case to conduct traceback investigations. The FDA conducted traceback for cases with limited cucumber exposure prior to illness onset, available purchase information, and often were geographically dispersed around the country. States also conducted traceback for additional clusters identified. An illness sub-cluster was defined as a single location or event where, (1) at least two or more people, who did not live in the same household, reported eating at the same restaurant location, attended a common event, or shopped at the same location of a grocery store and (2) became ill with the outbreak strain within seven days after their meal date and had meal dates within 10 days of one another. Other factors used in identifying case clusters for traceback included the following a reliable food history, places of purchase where more than one individual consumed product, high confidence of date(s) of exposure or purchase and case-patient clusters that were geographically dispersed whose onset dates spanned the outbreak period. The six traceback clusters were located in five different states, and included military facilities, restaurants and grocery stores. There were additional case-patient clusters that were not selected for FDA traceback analysis due to (1) the lack of epidemiologic information at the time that the traceback clusters were chosen, (2) the geographic areas of the case-patient exposures were already represented by other traceback clusters, or (3) the traceback clusters were located in similar geographic areas. The FDA, in collaboration with the CDC, reviewed the available epidemiologic information when the suspect vehicle was identified to determine the best sub-clusters of case-patients to trace back.

### Microbiological analysis and whole genome sequencing (WGS)

Foods suspected to be contaminated with the outbreak strains were sampled with standard bacterial analytical manual methods [8]. Investigations were performed to collect traceback information, standard operating procedures, customer lists and handling procedures at the implicated grower, a distribution centre, restaurants, grocery stores and import sites. The product was collected for testing throughout the distribution chain in the U.S. and the FDA also collected and tested other commodities grown by the same firm in Mexico that was identified as the supplier of contaminated cucumbers.

To further characterise relatedness between clinical and food isolates, WGS was performed on a subset of clinical isolates as well as all isolates recovered from the implicated product using techniques described by Crowe *et al*., [9]. WGS provides higher resolution than PFGE, allowing for more informative comparisons to be made between isolates. WGS allowed the evaluation of genetic relationships between the main outbreak strain and isolates exhibiting variant PFGE patterns.

CDC's National Antimicrobial Resistance Monitoring System (NARMS) laboratory conducted an antibiotic resistance test on isolates collected from six case-patients [10].

### Statistical analysis

An Epi Info (version 7.2.0.1) database was created and statistical analyses were performed using SAS version 9.3 (SAS Institute Inc, USA). The proportion of case-patients reporting consumption of specific foods in the week before illness onset was compared to the FoodNet Population Survey [7]. A binomial probability distribution was used to determine whether case-patients reported eating any foods at a significantly higher proportion than survey respondents. Demographics, exposure history and outcomes of case-patients with illness onset before and after product recall were compared. Reported food consumption, demographics, hospitalisation and death were analysed by standard methods between case-patients with illness onset before and after product recalls were issued.

### Epidemiologic studies

Two case-control studies of illness sub-clusters were conducted to identify foods associated with illness. Epidemiologists from the Minnesota Department of Health (MDH) recruited case-patients at locations of a national restaurant chain. Controls were well individuals who ate at the same locations in the same week. In addition, epidemiologists from the Department of Defense (DoD) completed a case-control study at a Marine Corps facility in California. Case-patients and controls who ate at a common dining facility were interviewed.

## Results

### Case-finding and patient demographics

**Fig. 1.**
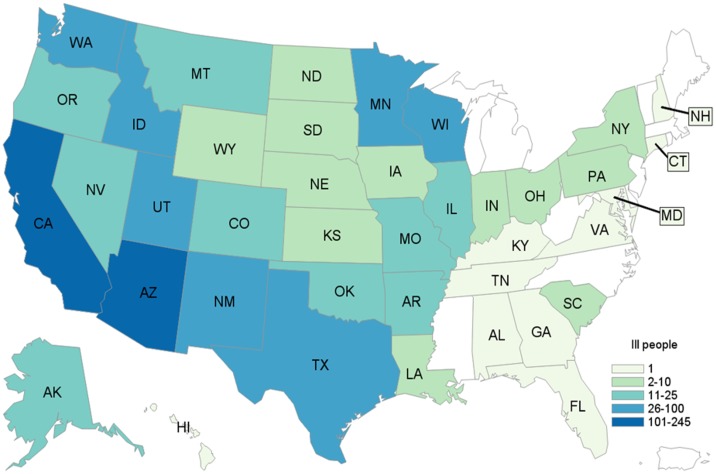
Distribution of case-patients due to outbreak strain of *Salmonella* Poona, by state. 3 July 2015–15 March 2016 (*n* = 907).

**Fig. 2.**
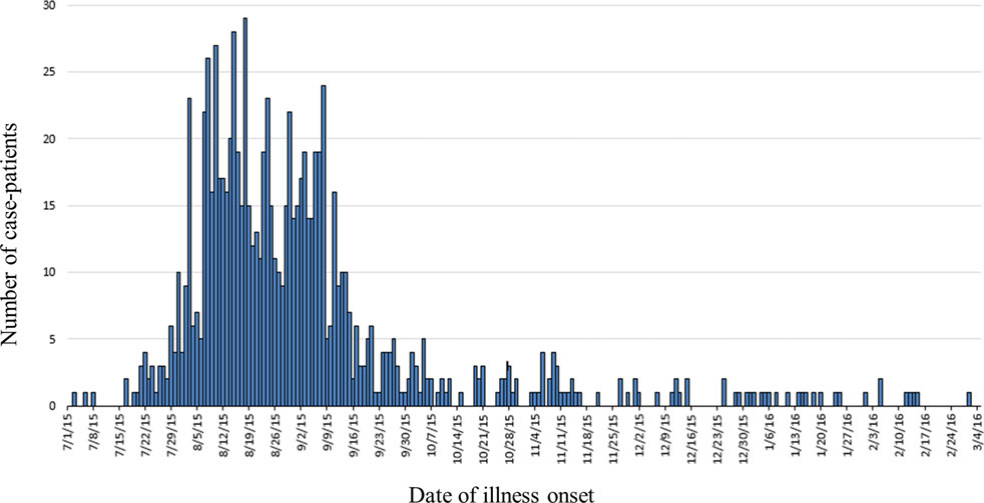
Epidemic curve of case-patients with the outbreak strain of *Salmonella* Poona, by date of illness onset, 3 July 2015–15 March 2016, United States (*n* = 907).

A total of 907 case-patients were identified from 40 states (Fig. 1). The highest number of case-patients were in California (*n* = 245) and Arizona (*n* = 140). Illness onset dates ranged from July 3, 2015 to March 2, 2016 (Fig. 2). The median age of patients was 18 years (range <1–99 years); 56% (502/893) were female. Twenty-two percent (144/661) were hospitalised; six deaths were reported from Arizona (1), California (3), Oklahoma (1) and Texas (1). *Salmonella* infection was not considered to be a contributing factor in two of the three deaths in California.

### Hypothesis generation

Data from 340 focused supplemental questionnaires from case-patients in 33 states were analysed. Sixty-three percent (215/340) of case-patients reported cucumber consumption in the seven days before illness onset. This proportion was significantly higher than in the 2006–2007 FoodNet Population Survey, in which 47% of healthy people reported consuming cucumbers in the seven days before interview (*p* < 0.001). No other food item was reported significantly higher than expected when compared to the FoodNet survey. Consultation with a group of produce industry experts early in the investigation validated the plausibility of cucumbers as the possible outbreak vehicle based on harvesting and distribution patterns.

### Microbiologic analysis

Between August 28, 2015 and December 3, 2015, over 75 cucumber samples were collected by federal and state partners from restaurants, grocery stores, distribution centres and at a United States–Mexico border import ports of entries. Nineteen cucumbers sourced from a single distributor (Distributor A) yielded the main outbreak strain in Arizona, California, Montana, Nevada, Utah and at the US–Mexico port of entry. All other food samples were negative for *Salmonella*.

All six isolates tested by NARMS laboratory were susceptible to all antibiotics tested [10].

### Whole genome sequencing

WGS analysis was completed for 154 clinical isolates, 14 with early onset date (July 3, 2015–August 2, 2015), 97 with onset date during peak of the outbreak (August 4, 2015–October 3, 2015) and 43 with late onset date (October 4, 2015–March 2, 2016). Nineteen isolates from cucumbers were analysed by WGS. All clinical and cucumber isolates with all five PFGE patterns analysed by WGS were closely related genetically, differing by 0–5 single nucleotide polymorphisms. A search of the National Center for Biotechnology Information (NCBI) database revealed a water sample, collected on September 9, 2015 by Mexico, was closely related genetically by WGS to the clinical and cucumber isolates in the outbreak.

### Epidemiologic studies

MDH officials enrolled 13 cases and 56 controls from five locations of a restaurant chain. Case-patients were significantly more likely than controls to report consumption of a garden salad [odds ratio (OR) = 12.0 (*p* < 0.0005)]. The garden salad contained a lettuce mix, cucumbers, grape tomatoes, red onion and croutons. All cucumbers served at the restaurant were sourced from Distributor A. The garden salad was the only menu item that contained cucumbers, lettuce mix and grape tomatoes, limiting the utility of an ingredient-specific analysis.

DoD epidemiologists enrolled seven cases and 14 controls from a single dining facility. Case-patients were significantly more likely than controls to report consumption of a lettuce and cucumber mix [OR = 14 (*p* < 0.005)]. All cucumbers served at the dining facility were also sourced from Distributor A. Cucumbers were only served mixed with lettuce, thus preventing ability to analyse individual ingredients. No other menu item was associated with the case status in either study.

### Traceback investigation

**Fig. 3.**
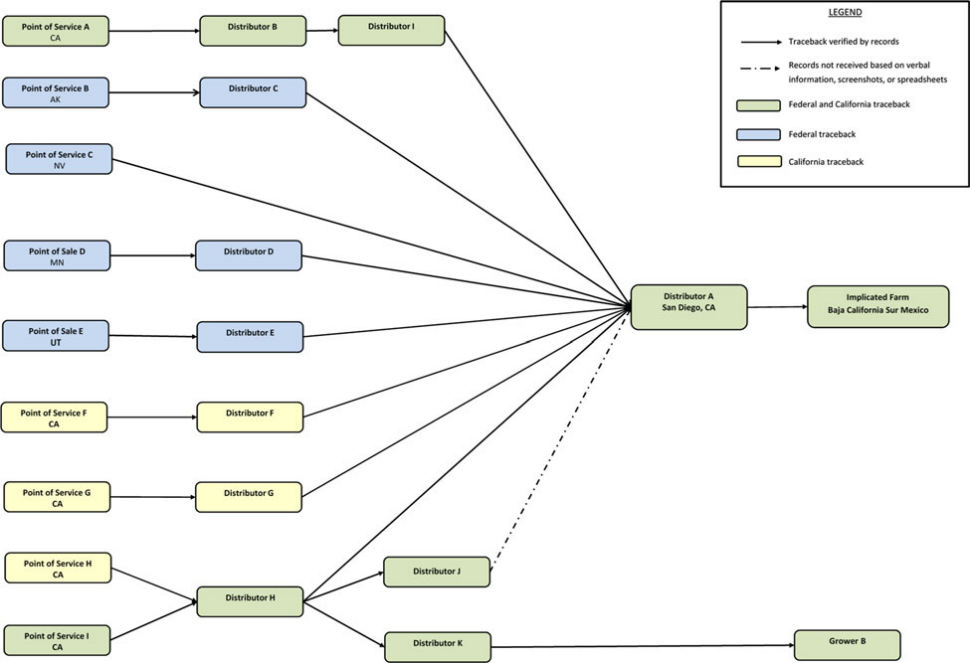
Traceback diagram for multistate outbreak of *Salmonella* Poona infections associated with imported cucumbers investigation.

Interviews of case-patients revealed ten illness sub-clusters. Six sub-clusters were chosen by the FDA to investigate based on quality and quantity of available information as well as geographic distribution of cases. California public health officials traced back two additional sub-clusters and one single case with exposure in California that were not included in the FDA traceback. Results indicated that cucumbers consumed by case-patients were imported from Mexico and distributed by Distributor A. Traceback investigations revealed that Distributor A, located in San Diego, California, sourced cucumbers to national grocery stores and restaurant chains. Further investigation revealed that all implicated cucumbers sold by Distributor A were grown by a single grower in Baja California Sur, Mexico (Fig. 3).

### Control measures/recall

On September 4, 2015, Distributor A voluntarily recalled all cucumbers sold under a single brand label from August 1, 2015, to September 3, 2015. The CDC and FDA issued public web announcements describing the investigation and providing advice to consumers. On September 6, 2015, the DoD issued an All Food and Drug Acts (ALFOODACT) message informing all military installations not to serve cucumbers from Distributor A.

On September 14 and 23, 2015, the implicated farm was added to two FDA Import Alerts [11,12] due to the presence of pathogenic contamination. Cucumber samples yielding the outbreak strain, case exposure information and traceback investigation were used as evidence to support the issuance of these Import Alerts.

Illnesses were first reported on July 3, 2015 and peaked in mid-August. Despite product recalls, cases continued to be reported for an additional seven months after the implicated cucumbers were recalled. To examine whether or not the same source of contamination was responsible for the illnesses reported after the recall, we compared food histories and demographics of those who became ill before and after the recalls were issued. Due to the shelf life of cucumbers, post-recall case-patients were defined as those whose illness onset occurred later than three weeks after the final recall was issued on September 11, 2015. Four hundred sixty-nine case-patients were identified with exposure dates after this date. The reported cucumber consumption was not significantly different, 65% *vs.* 60%, between these groups. Consumption of other produce items, like tomatoes, was also analysed by pre- and post-recall groups (Table 1). Furthermore, the age range, and gender proportion of ill individuals did not differ significantly before and after the recalls. The FDA and state partners continued to collaborate on sampling of the product and investigating possible routes of cross contamination in the supply chain. The FDA sampled other imported commodities from the implicated farm, customers of the packinghouse owned by the implicated farm and product identified by traceback after the recall.

**Table 1.** Frequency of selected food exposures in case-patients with outbreak-associated illnesses *vs*. 2006 FoodNet population survey as of 2 March 2016

	Pre-recall	Post-recall	Total	Expected from FoodNet Population Survey^a^
Any cucumber consumption (# of responses)^b^	65.1% (132/212)	60.2% (77/128)	63.2% (215/340)	46.9%
Any tomato consumption (# of responses)^b^	48.3% (86/178)	35.6% (48/135)	42.8% (134/313)	59.6%

a Foodborne Diseases Active Surveillance Network (FoodNet) Population Survey Atlas of Exposures, 2006–2007.

b Any consumption in the 7 days before illness onset.

### Grower inspection/root-cause analysis

Inspections of the grower and packing facility in Mexico were completed by the FDA, with Mexican Food Safety Authorities' collaboration (Servicio Nacional de Sanidad, Inocuidad y Calidad Agroalimentaria (SENASICA) and Comisión Federal para la Protección contra Riesgos Sanitarios (COFEPRIS)). Potential sources and routes of contamination were noted, including concerns with waste water-management, equipment design of the flume handling system area and storage of packing materials. However, no samples were collected and the root-cause of the outbreak was not determined.

## Discussion

This outbreak of *Salmonella* Poona infections resulted in 907 reported illnesses. It was the largest US foodborne disease outbreak in the last ten years and the third largest in the past 20 years [13]. The outbreak grew rapidly, expanding from 18 illnesses reported at the time of detection to 305 illnesses reported at the time of recall 17 days later. More than 600 cases were identified after the date of the first product recall. Additionally, the number of people potentially exposed to the implicated cucumbers was high, as they were sold at large national restaurant chains as well as at the two largest retailers in the United States [14]. Collaboration between state, local and federal public health agencies using epidemiological and traceback information resulted in the rapid identification of the outbreak vehicle and subsequent regulatory action to mitigate the impact of the outbreak.

Cucumbers were recalled in this outbreak. The short time from outbreak detection to collection of cucumbers that yielded the outbreak strain allowed for recalls to be issued while the contaminated cucumbers were still available for purchase. Identification of illness sub-clusters in this outbreak was important in identifying the distributor and grower quickly. Also, product sampling by state and federal regulatory officials yielded the outbreak strain.

Despite swift identification of the outbreak vehicle and issuance of recalls, additional cases were identified after product action was taken. Based on the shelf life of cucumbers, we expected all cucumbers sold before the recalls to be off the market (and therefore unavailable for consumption) by September 25, 2015. However, over half of the total number of case-patients associated with this outbreak reportedly ate cucumbers after that date. Epidemiologic, traceback and laboratory analyses support the conclusion that all illnesses, including the late onset illnesses, were linked to the same source. Possible explanations for additional illnesses occurring after the recalls were implemented, include (1) cross-contamination of other food products that remained available for consumption after September 25, 2015, (2) inadequate sanitisation of reusable plastic containers or grocery bags used to hold contaminated cucumbers and (3) persistent environmental contamination with *Salmonella* at produce packing houses or distribution sites. To prevent contaminated cucumbers from entering the United States from other shippers in Mexico, the FDA increased surveillance on cucumbers imported from Mexico. The FDA also sampled other commodities and monitored subsequent cucumber recalls after the initial recall and Import Alerts to ensure that the cucumbers were not contaminated with the outbreak strain of *Salmonella*. The low level of genomic diversity observed in this outbreak is consistent with other point source *Salmonella* outbreaks that have been characterised using PFGE and WGS, such as *Salmonella* Montevideo in salami [15] and *Salmonella* Enteritidis in eggs [16]. Given the short shelf life (2 weeks) of fresh cucumbers, it is extremely unlikely that illnesses observed 3 or 4 months after the onset of the outbreak reflect exposure to the contaminated product. Unfortunately, there is limited information on the role that cross-contamination plays in the origin or continuation of foodborne illness outbreaks [17]. However, it has been well established that *Salmonella* spp. can survive on food contact surfaces for a prolonged period, thus increasing the risk of cross-contamination between food products and food contact surfaces [18,19]. Ultimately, a cause for the substantial number of post-recall cases was not identified.

Cucumbers are becoming increasingly recognised as a common vehicle for *Salmonella* outbreaks in the United States. The first identified multistate outbreak of cucumber-associated *Salmonella* infections occurred in 2009. Since 2013, there have been at least four other recognised multistate outbreaks of *Salmonella* infections linked to the consumption of contaminated cucumbers in the United States [20–22]. This outbreak was the largest US foodborne disease outbreak in the last ten years and the third largest in the past 20 years. In response to this outbreak, multiple public health agencies collaborated in a concerted and expedient effort to remove a fresh produce item from commerce. Despite the large number of case-patients identified after the cucumbers were recalled, it is likely that a delay in product action would have resulted in a contaminated product being available longer, resulting in an even greater number of illnesses.

## Disclaimer

The findings and conclusions in this report are those of the authors and do not necessarily represent the official position of CDC.
